# Trends in nutritional status and factors affecting prognostic nutritional index in ovarian cancer patients during chemotherapy: a prospective longitudinal study based on generalized estimating equations

**DOI:** 10.1007/s00520-024-08384-8

**Published:** 2024-02-24

**Authors:** Zhang Mengqin, He Yalin, Liu Xing, Liu Ya, Tian Yalin, Dan Xin, Ren Jianhua

**Affiliations:** 1 Department of Gynecological Nursing, West China, Second University Hospital, Sichuan University/West China School of Nursing, Sichuan University, Chengdu, Sichuan China; 2 Key Laboratory of Birth Defects and Related Diseases of Women and Children (Sichuan University), Ministry of Education, Chengdu, Sichuan China; 3 Department of Nursing, West China Second University Hospital, Sichuan University, Chengdu, Sichuan China

**Keywords:** Ovarian cancer, Chemotherapy, Prognostic nutritional index, Nutritional status, Nutritional risk

## Abstract

**Purpose:**

Numerous studies have investigated the relationships between nutritional status and the prognosis of ovarian cancer (OC). However, the majority of these studies have focused on pre-chemotherapy malnutrition, with limited attention given to dynamic changes in nutritional status during chemotherapy and the associated risk factors affecting the prognostic nutritional index (PNI) in OC women. This study aims to explore the variation trend in the nutritional status of OC women over time during chemotherapy and assess its predictive factors.

**Methods:**

A prospective longitudinal study was conducted from January 2021 to August 2023. Body mass index (BMI), PNI, Nutritional Risk Screening (NRS) 2002, serum albumin, and prealbumin measurements were utilized to assess the nutritional status of OC women. Data were collected through face-to-face interviews before initial chemotherapy (T0) and during the first (T1), third (T2), and fifth (T3) cycles of chemotherapy. Generalized Estimating Equations (GEE) were employed for the analysis of potential predictive factors.

**Results:**

A total of 525 OC women undergoing chemotherapy completed the study. Significantly varied levels of BMI, PNI, and serum concentrations of hemoglobin, albumin, prealbumin, potassium, sodium, magnesium, and calcium were observed in these patients (*p* < 0.05). The prevalence of nutritional risk decreased over time during chemotherapy (*p* < 0.05). Nutritional parameters, including BMI, PNI, and the serum concentrations of albumin and prealbumin, exhibited an upward trend in nutritional status throughout the chemotherapy cycles (*p* < 0.05). Multivariate analysis indicated that higher levels of BMI, serum albumin, prealbumin, absolute lymphocyte count, and hemoglobin ≥ 110 g/L at admission were associated with elevated PNI after chemotherapy (*β* = 0.077, *p* = 0.028; *β* = 0.315, *p* < 0.001; *β* = 0.009, *p* < 0.001; *β* = 1.359, *p* < 0.001; *β* =  − 0.637, *p* = 0.005).

**Conclusion:**

Patients consistently demonstrated improvements in nutritional risk and status from the initiation to the completion of chemotherapy cycles. Nutritional monitoring of OC women, particularly those exhibiting abnormalities at the commencement of chemotherapy, is crucial. Targeted nutritional support programs should be developed to enhance the prognosis of OC women.

**Supplementary Information:**

The online version contains supplementary material available at 10.1007/s00520-024-08384-8.

## Introduction

Ovarian cancer (OC) stands as one of the most prevalent gynecological malignancies, ranking as the fifth leading cause of cancer-related deaths among women in the USA [1]. Globally, 313,959 women were diagnosed, with 207,252 succumbing to OC in 2020 [2]. In China, it was estimated that there were 55,342 new cases of ovarian cancer and 37,519 deaths in 2020 [3], and a rising occurrence has been observed in recent years [4]. The standard treatment for ovarian cancer involves surgery followed by platinum/paclitaxel-based chemotherapy, typically extending over multiple cycles (6-8). Compared to other gynecological cancers, OC patients face a heightened risk of malnutrition [5]. Studies reveal that a majority of OC patients undergoing surgery experience moderate or severe malnutrition [6], with post-operative chemotherapy exacerbating malnourishment in 76.1% of cases [7].

The etiology of malnutrition in cancer patients is often multifaceted, involving factors such as inadequate dietary intake, lack of physical activity, and metabolic disturbances, leading to systemic inflammatory responses with direct impacts on metabolic pathways [8]. While chemotherapy is a common strategy for advanced cancer treatment, it brings about detrimental side effects, including gastrointestinal symptoms, sore mouth, and loss of appetite, resulting in compromised nutritional status. Malnutrition has been correlated with a diminished quality of life, extended hospital stays, and reduced survival rates in cancer patients [9, 10]. Especially for OC women, malnutrition is associated with increased readmissions, reoperations, and complications [11]. Gupta et al. [12] reported that OC women with improved nutritional status exhibited significantly better survival rates than those with deteriorating nutritional status. Despite the importance of early prevention and detection of malnutrition, these aspects are frequently overlooked during chemotherapy. Therefore, identifying appropriate prognostic biomarkers to effectively assess nutritional and immune status is crucial, holding significant promise for enhancing the prognosis of OC women.

Initially proposed by Onodera et al. [13], the Prognostic Nutritional Index (PNI) stands out as one of the most frequently utilized nutritional parameters for evaluating nutritional status. Originally, designed to assess the nutritional status of surgical patients, the PNI has evolved in recent years to predict post-operative complications and the long-term progression of various malignant tumors. It has also emerged as a valuable prognostic indicator for lung cancer, gastrointestinal cancer, and gynecological cancer patients [14]. Calculated through a simple formula utilizing only serum albumin levels and absolute lymphocyte cell count in peripheral blood, the PNI has become an integral tool in nutritional assessment.

Multivariate analysis has identified a low PNI as a significant independent predictor of poor prognosis in OC women. Notably, a low PNI level has been correlated with shorter overall survival (OS) [15], progression-free survival (PFS) [15–17], and platinum resistance [17, 18] in OC women. Despite its pivotal role in influencing survival outcomes, few studies have explored the influencing factors of the nutritional index in cancer patients, particularly in OC women. However, Chen et al. [19] reported a significant association between a high PNI and serum albumin and lymphocytes in breast cancer patients undergoing chemotherapy.

To our knowledge, most studies on the nutritional status and risk of OC patients are cross-sectional, neglecting the variation tendency of nutritional risk during chemotherapy in longitudinal research. This gap in research hinders health professionals from gaining a comprehensive understanding and taking effective management of nutritional risk during chemotherapy. Therefore, our study aimed to address this gap by conducting a longitudinal investigation. The primary objective was to explore the dynamic changes in nutritional status during chemotherapy and identify relevant risk factors influencing the prognostic nutritional index in OC women.

## Methods

### Study design

This prospective longitudinal study spanned from January 2021 to August 2023. We enrolled ovarian cancer (OC) patients scheduled for post-operative adjuvant chemotherapy at West China Second University Hospital, Sichuan University. Chemotherapy, comprising a minimum of six cycles spaced 21 days apart, was determined based on clinical stage and patient condition. Common regimens included TP (paclitaxel and cisplatin), TC (paclitaxel with carboplatin), BEP (bleomycin, etoposide, and cisplatin), IP (irinotecan and cisplatin), GP (gemcitabine and cisplatin), TL (paclitaxel and oxaliplatin), T (paclitaxel), and FOLFOX (folinic acid, 5-fluorouracil, and oxaliplatin). The regimen selection was at the discretion of physicians. Data collection involved face-to-face interviews at initial chemotherapy (T0), and on the first (T1), third (T2), and fifth (T3) cycles.

### Patients and variables

Inclusion criteria comprised OC diagnosis, age 18 and above, prior gynecological surgery, Chinese language proficiency, mental acuity to participate, and informed consent. Exclusion criteria included prior or ongoing inflammatory diseases. Patients with incomplete study participation and those with a treatment delay of ≥ 2 weeks due to chemotoxicity were excluded from data analysis.

Nutritional parameters included PNI, BMI, serum albumin, and prealbumin. Blood tests were conducted pre- and post-chemotherapy, with pre-tests taken within 1 week before chemotherapy commencement and post-tests obtained 1 week after discharge. PNI values were calculated as 10 × serum albumin (g/dL) + 0.005 × absolute lymphocyte count (cells/mm^3^) [13].

The Nutritional Risk Screening (NRS) 2002, established by the European Society for Parenteral and Enteral Nutrition (ESPEN), was employed for nutritional risk screening, with a total value ≥ 3 indicating nutritional risk [20].

Data encompassed potential predictive factors for PNI across three domains: social-demographic characteristics, clinical characteristics, and blood test results. Social-demographic factors included age, nationality, marital status, education level, habitation, monthly income, and BMI. Clinical factors embraced diabetes complications, restorative intestinal resection, change in dietary intake, daily sleep duration, defecation frequency, regular bowel habits, daily activity steps, chemotherapy regimen, chemotherapy-related nausea and vomiting, Karnofsky performance scale (KPS) score, Barthel index, in-hospital nutritional support, in-hospital parenteral and enteral nutritional support, in-hospital blood transfusion, and hospital days. Blood test results encompassed serum levels of albumin, prealbumin, hemoglobin, absolute lymphocyte count, serum potassium, sodium, magnesium, calcium, absolute lymphocyte count, and fasting blood glucose.

#### Definition of clinical variables

Some clinical variables are defined below.

Change in dietary intake was evaluated by a food frequency questionnaire or dietary recall interview, and calculated at the estimating difference between baseline daily intake before the initial chemotherapy treatment and the mean of intake during the preceding month. Change in dietary intake was divided into five categories: unchanged, increase (≤ 50%), increase (> 50%), reduce (≤ 50%), and reduce (> 50%).

Daily sleep duration was self-reported in response to the question: “How many hours do you sleep on average per day?” Sleep duration was classified into two categories: short sleep (< 7 h/day) and normal sleep duration (≥ 7 h/day). Daily activity steps were extracted directly from the average daily count calculated by the mobile phone to measure activity levels during the intervals of chemotherapy.

Defecation frequency was the average number of stools per week during the intervals of chemotherapy, with three categories of ≤ 3 times/week, 4–7 times/week, and ≥ 8 times/week. Regular bowel habit was assessed by the following question: “Do you develop regular bowel habits during the intervals of chemotherapy?” (regular or irregular).

In-hospital nutritional support was defined as parenteral nutrition or enteral nutrition support during hospitalization. Any intravenous infusion of albumin, amino acids, and fat emulsion during hospitalization was defined as parenteral nutrition support, which was determined by clinicians’ concerns for the nutritional conditions and dietary intake of the patients. Chemotherapy-induced hyperemesis was the indication of parenteral nutrition support. In-hospital enteral nutrition was defined as any intake of protein powder supplements, nutrient powder supplements, and other fortified complementary foods depending on the patient’s preference or clinicians’ advice during hospitalization.

### Data collection

Data were systematically gathered by trained registered nurses in the gynecological chemotherapy ward, having undergone uniform training beforehand. Collection occurred at admission, discharge, and within the week post-discharge for each specified chemotherapy cycle (T1, T2, and T3).

Social-demographic characteristics, weekly habits (diary, activity, and bowel habits), and pre-chemotherapy blood test results were obtained through face-to-face interviews or electronic medical records at admission. Information regarding chemotherapy-induced nausea and vomiting, nutritional supports (parenteral and enteral), blood transfusion during hospitalization, post-chemotherapy performance status score, and hospital days were acquired at discharge. Post-chemotherapy blood test results were obtained through telephone follow‐up in the week following discharge. Weekly routine re-examinations covering blood routine, urine, and liver and kidney function were conducted.

Ethical approval was secured from the local ethics committee with ethical No. of 2,021,190.

### Statistical analysis

Inclusion in data analysis required complete data for all three follow-ups. Categorical variables were presented as frequency (*n*) and proportion (%), while continuous variables were described using mean and standard deviation (SD) or median (interquartile). The Chi-square test, independent *t*-test, and one-way repeated measures ANOVA were employed for the comparisons of baseline and clinical variables between time points, as well as between those completing entire follow-up and those who were not. The Kruskal–Wallis *H* test was conducted to compare the KPS score and Barthel index.

Univariate and multivariate Generalized Estimating Equation (GEE) analyses, employing an autocorrelation working correlation matrix, were conducted to identify significant factors contributing to PNI. All available data, including data from patients lost to follow-up, were included in the GEE analyses. Variables with a *p*-value below 0.1 in univariate analyses were included in multivariate analyses.

Statistical analyses utilized the Statistic Package for Social Science software (SPSS) ver.23. Post hoc analysis of pairwise comparisons was Bonferroni-corrected based on the pre-determined number of comparisons (with a corrected *α* of 0.05/3 = 0.017; 0.05/6 = 0.008). The significance level for all analyses was set at 0.05.

## Results

### Patient characteristics

**Fig. 1:**
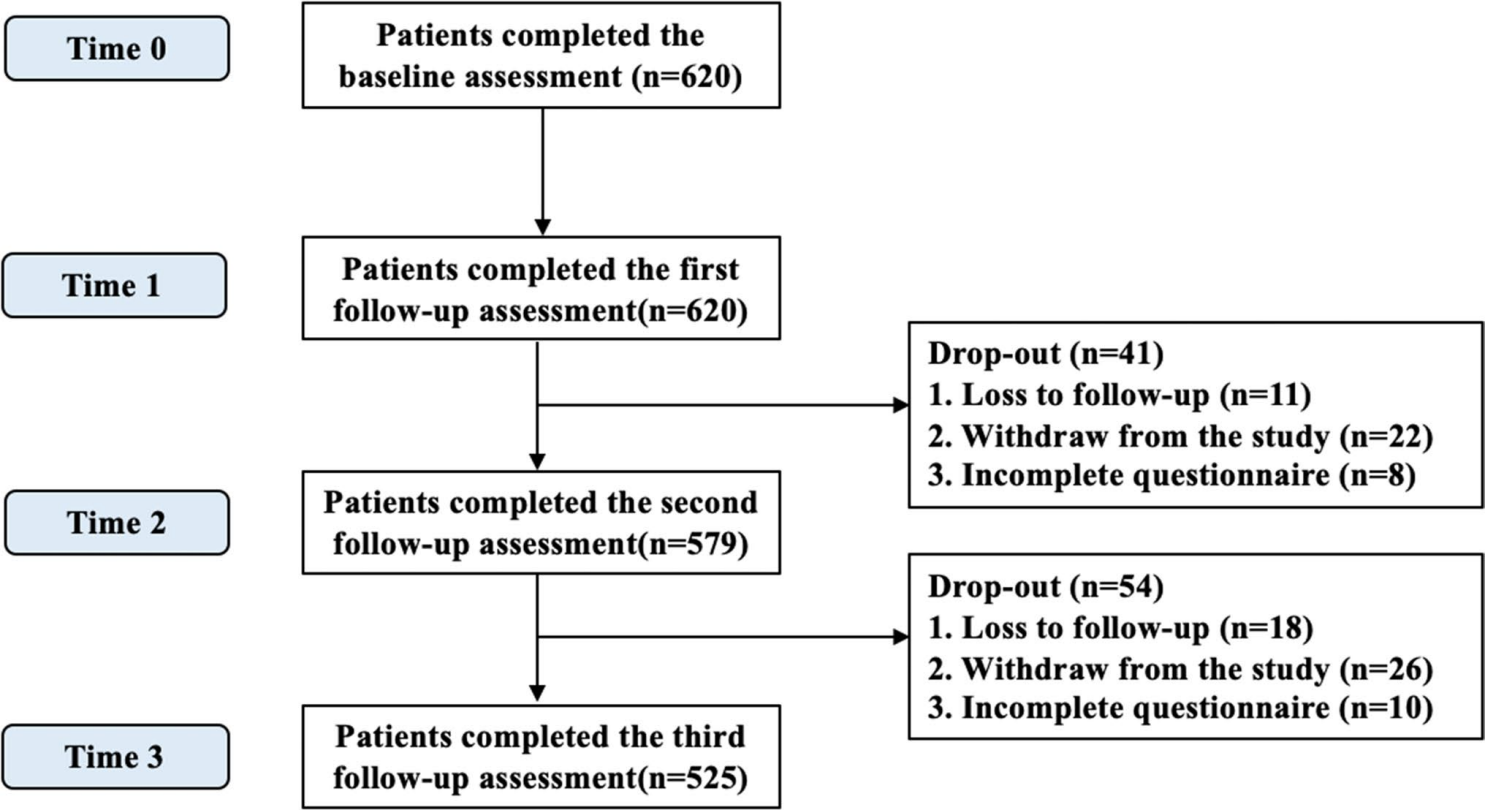
Flowchart of the study participants over the study period

A total of 620 patients were initially recruited, and 525 (84.7%) successfully completed the entire study (Fig. 1). Ninety-five (15.3%) women were lost to follow-up, mainly due to the patients terminating the chemotherapy treatment as a result of serious adverse reactions, or transferring to other hospitals because the delay to obtain an appointment for chemotherapy was too long. There were no significant differences in baseline characteristics between the women lost to follow-up and those who were not (see supplementary Table 1, Additional file).

Among the 525 patients, the average age was 52.85 (SD = 10.67) years, with 15.0% aged above 65. Predominantly, the patients were of Han Chinese ethnicity (97.9%), married (86.5%), and possessed a lower education level (55.1% with junior school or below). More than half resided in urban areas (56.6%), and 64.0% reported a per capita monthly income exceeding ¥3001. A small percentage had diabetes (8.8%), while 15.6% underwent intestinal resection. Detailed characteristics are summarized in Table 1.

**Table 1:** Baseline characteristics of participants (*N* = 525)

Variables	*N*	%
Age (year)		
Means ± SD^a^	52.85 ± 10.67	
≤ 35	33	6.3
36–64	413	78.7
≥ 65	79	15
Nation		
Han	514	97.9
Other	11	2.1
Marital status		
Married	454	86.5
Unmarried	20	3.8
Divorced	24	4.6
Widowed	27	5.1
Educational level		
Primary school or below	138	26.3
Junior high school	151	28.8
Senior high school	95	18.1
Junior college	78	14.9
Undergraduate or above	63	12.0
Habitation		
Rural	92	17.5
Town	136	25.9
Urban	297	56.6
Per capita monthly income (yuan)		
≤ 630	16	3.0
631 ~ 1000	40	7.6
1001 ~ 3000	133	25.3
3001 ~ 5000	229	43.6
5001 ~ 10,000	81	15.4
> 10,000	26	5.0
Complication of diabetes		
Without	479	91.2
With	46	8.8
Intestinal resection		
Without	443	84.4
With	46	15.6

^a^Standard deviation

### Changes in nutritional risk during chemotherapy

Table 2 outlines the shifts in nutritional risk during chemotherapy. The prevalence of nutritional risk at times 0, 1, 2, and 3 were 17.0%, 14.7%, 7.4%, and 5.1%, respectively, indicating a decreasing trend in nutritional risk prevalence among OC women as chemotherapy progressed.

**Table 2:** Changes in nutritional risk during chemotherapy

	Nutritional risk^a^ (*N*, %)		*χ*^2^
	Yes	No	
T0	89 (17.0)	436 (83.0)	51.248**
T1	77 (14.7)	448 (85.3)	
T2	39 (7.4)	486 (92.6)	
T3	27 (5.1)	498 (94.9)	

^a^Nutritional risk was assessed upon discharge on each specified chemotherapy cycle (T1, T2, and T3)

**p < 0.001

### Variation trends in nutritional and hematological levels over time during chemotherapy

Table 3 presents statistically significant differences from baseline through follow-up in BMI, serum albumin, serum prealbumin, PNI, hemoglobin, serum potassium, serum sodium, serum magnesium, and serum calcium (*p* < 0.05). No statistically significant differences were observed in absolute lymphocyte count, serum chloride, and fasting blood glucose.

**Table 3:** Variation trends in nutritional and hematological levels over time during chemotherapy (mean ± standard deviation)

Variables^#^	T0	T1	T2	T3	*F*^&^
BMI at admission (kg/m^2^)	—	22.30 ± 3.15	22.43 ± 3.08	22.57 ± 3.13	15.090**
Albumin (g/L)	42.39 ± 4.19	43.23 ± 3.14^a^	43.74 ± 3.14^a^	43.86 ± 3.28^ab^	27.140**
Prealbumin (mg/L)	214.38 ± 59.53	221.84 ± 54.14	240.08 ± 51.61^ab^	238.55 ± 53.61^ab^	49.058**
Absolute lymphocyte count (× 10^9^/L)	1.48 ± 0.89	1.48 ± 0.54	1.49 ± 0.53	1.42 ± 0.53	2.022
PNI	49.77 ± 6.20	50.61 ± 4.21	51.22 ± 4.23^a^	50.96 ± 4.26^a^	12.676**
Hemoglobin (g/L)	117.43 ± 13.56	113.71 ± 12.52^a^	111.31 ± 13.30^ab^	107.86 ± 13.94^abc^	106.251**
Serum potassium (mmol/L)	4.16 ± 0.36	4.09 ± 0.39^a^	4.02 ± 0.35^ab^	4.00 ± 0.37^ab^	29.228**
Serum sodium (mmol/L)	140.33 ± 2.47	140.48 ± 3.17	140.53 ± 3.29	140.98 ± 2.93^a^	5.123*
Serum chloride (mmol/L)	105.43 ± 3.02	105.85 ± 4.08	105.79 ± 3.91	106.09 ± 4.82	2.574
Serum magnesium (mmol/L)	0.79 ± 0.10	0.76 ± 0.13^a^	0.69 ± 0.12^ab^	0.65 ± 0.12^abc^	237.408**
Serum calcium (mmol/L)	2.27 ± 0.15	2.31 ± 0.16^a^	2.29 ± 0.14	2.30 ± 0.17	6.707**
Fasting blood glucose (mmol/L)	5.57 ± 1.05	5.56 ± 1.11	5.54 ± 1.12	5.56 ± 1.15	0.180

^a^Compared with T0, *p* < 0.05/6 = 0.0083

^b^Compared with T1, *p* < 0.05/6 = 0.0083

^c^Compared with T2, *p* < 0.05/6 = 0.0083

^#^The nutritional and hematological variables were assessed in the week after discharge

^&^Greenhouse–Geisser correction was used when the assumption of sphericity was not met

**p < 0.0005, * *p* = 0.002

Regarding nutritional parameters, both BMI and serum albumin levels increased over time. For prealbumin and PNI, a slight increase was noted from T0 to T2 (*p* < 0.05/6 = 0.0083), followed by a slight decrease from T2 to T3 (*p* > 0.05). Compared to T0, post hoc analysis of pairwise comparisons revealed significant increases in serum albumin, prealbumin, and PNI levels at T2 and T3 (*p* < 0.05/6 = 0.0083). Similarly, BMI significantly increased at T2 and T3 compared to T1 (*p* < 0.05/3 = 0.0167). The variation trend in nutritional parameters indicated an upward trend in nutritional status with the progression of chemotherapy.

Regarding serum electrolyte levels, Table 3 indicates that both serum potassium and serum magnesium levels decreased over time, while serum sodium levels increased. Pairwise comparisons showed a significant decrease in both serum potassium and serum magnesium at T2 and T3 compared to T0.

### Determinants for variation of PNI in ovarian cancer patients

We employed the GEE model to scrutinize the influential factors of PNI in 620 OC patients. In the univariate analysis, focusing on individual variables, several factors exhibited significant associations with PNI (Table 4). These included BMI measured at admission, habitation, defecation frequency, regular bowel movement in the past week, pre-chemotherapy anemia, serum albumin level at admission, prealbumin level at admission, absolute lymphocyte count at admission, chemotherapy regimen during hospitalization, Barthel index after chemotherapy, blood transfusion during hospitalization, and the hospital days.

**Table 4:** Univariate and multivariate generalized estimation equation analysis of factors predicting the prognostic nutritional index (PNI) of ovarian cancer patients received chemotherapy

Variables	Univariate analysis			Multivariate analysis		
	*β*	95% CI	*p* value	*β*	95% CI	*p* value
Age (year)						
≤ 35	Ref			Ref		
36–64	− 1.148	− 2.227– − 0.069	0.037	− 0.743	− 1.606–0.120	0.091
≥ 65	− 1.544	− 2.854– − 0.233	0.021	− 0.661	− 1.727–0.404	0.224
BMI at admission (kg/m^2^)	0.127	0.044–0.209	0.003	0.077	0.008–0.146	0.028
Nation						
Han	Ref					
Other	− 0.290	− 1.754–1.175	0.698			
Marital status						
Married	Ref					
Unmarried	1.215	0.016–2.413	0.047			
Divorced	− 0.479	− 1.744–0.785	0.457			
Widowed	− 0.193	− 1.305–0.920	0.734			
Educational level						
Primary school or below	Ref					
Junior high school	− 0.188	− 0.650–0.489	0.670			
Senior high school	0.257	− 0335–1.007	0.412			
Junior college	0.433	− 0.332–1.198	0.324			
Undergraduate or above	0.423	− 0.587–1.219	0.534			
Habitation						
Rural	Ref					
Town	− 0.666	− 1.379–0.048	0.068			
City	− 0.256	− 0.896–0.384	0.433			
Per capita monthly income (yuan)						
≤ 630	Ref					
631 ~ 1000	− 0.612	− 2.828–1.604	0.588			
1001 ~ 3000	− 0.352	− 2.470–1.766	0.745			
3001 ~ 5000	0.241	− 1.846–2.328	0.821			
5001 ~ 10,000	0.436	− 1.739–2.610	0.695			
> 10,000	− 0.412	− 2.814–1.989	0.736			
Complication of diabetes						
Without	Ref					
With	− 0.037	− 1.041–0.967	0.942			
Intestinal resection						
Without	Ref					
With	− 0.485	− 1.164–0.194	0.162			
Change in dietary intake^a^						
Unchanged	Ref					
Increase (≤ 50%)	− 0.100	− 1.159–0.959	0.853			
Increase (> 50%)	− 1.008	− 3.260–1.244	0.380			
Reduce (≤ 50%)	− 1.462	− 3.422–0.498	0.144			
Reduce (> 50%)	− 1.296	− 3.152–0.560	0.171			
Average daily sleep duration (h)						
≥ 7	Ref					
< 7	− 0.368	− 0.844–0.107	0.129			
Defecation frequency(times/week)						
0–3	Ref			Ref		
4–7	2.453	0.368–4.538	0.021	2.228	0.226–4.231	0.029
≥ 8	2.510	0.408–4.612	0.019	2.416	0.404–4.428	0.019
Regular bowel habit						
With	Ref			Ref		
Without	− 1.280	− 1.880– − 0.159	0.029	− 0.274	− 0.932–0.384	0.414
Average daily activity steps						
< 4400	Ref					
4400 ~ 7500	0.189	− 0.294–0.672	0.444			
> 7500	0.093	− 0.623–0.810	0.798			
Pre*-*chemotherapy anemia^b^						
Without	Ref			Ref		
With	− 1.724	− 2.195– − 1.1254	< 0.001	− 0.637	− 1.079– − 0.195	0.005
Serum albumin (g/L)	0.418	0.338–0.499	< 0.001	0.315	0.228–0.401	< 0.001
Serum prealbumin (mg/L)	0.020	0.016–0.025	< 0.001	0.009	0.004–0.013	< 0.001
Absolute lymphocyte counts (× 10^9^/L)	1.494	0.898–2.091	< 0.001	1.359	0.868–1.850	< 0.001
Chemotherapy regimens^c^						
TP	Ref			Ref		
TC	0.258	− 0.361–0.877	0.414	0.297	− 0.220–0.815	0.054
Others	− 1.604	− 2.617– − 0.591	0.002	− 1.188	− 1.980– − 0.396	0.003
Chemotherapy-induced nausea^d^						
Grade < 2	Ref					
Grade ≥ 2	− 0.058	− 0.511–0.395	0.801			
Chemotherapy-induced vomiting^e^						
Grade < 2	Ref					
Grade ≥ 2	− 0.200	− 0.756–0.356	0.481			
KPS score^f^	0.032	− 0.088–0.153	0.599			
Barthel index	0.051	0.017–0.085	0.004	− 0.003	− 0.034–0.027	0.833
In-hospital nutritional support^g^						
Without	Ref					
With	− 0.881	− 1.936–0.175	0.102			
In-hospital parenteral nutritional support						
Without	Ref			Ref		
With	− 1.432	− 3.070–0.205	0.086	− 0.220	− 1.549–1.110	0.746
In-hospital enteral nutritional support						
Without	Ref					
With	− 0.299	− 1.414–0.817	0.600			
In-hospital blood transfusion						
Without	Ref			Ref		
With	− 6.476	− 10.929– − 2.022	0.004	− 4.695	− 9.873–0.483	0.076
Length of hospital stay	− 0.172	− 0.295– − 0.050	0.006	0.074	− 0.057–0.204	0.268

*Ref.*: reference

^a^Dietary intake during the preceding month

^b^Hemoglobin < 110 g/L in women

^c^Chemotherapy regimens: TP regimen: contained paclitaxel with cisplatin; TC regimen: contained paclitaxel with carboplatin; others included BEP (bleomycin, etoposide, and cisplatin), IP (irinotecan and cisplatin), GP (gemcitabine and cisplatin), TL (paclitaxel and oxaliplatin), T (paclitaxel), and FOLFOX (folinic acid, 5-fluorouracil and oxaliplatin)

^d,e^Toxicity was graded according to Common Terminology Criteria for Adverse Events (CTCAE, v5)

^f^Karnofsky performance scale, KPS

^g^Parenteral and enteral nutritional support, including intravenous infusion of albumin and amino acids and nutrition intake of protein powers, nutrient powders, and other fortified complementary foods

Upon incorporating all significant factors into the multivariate GEE model, seven factors emerged as independent predictors of PNI after chemotherapy (Table 4). Higher BMI, serum albumin, prealbumin, and absolute lymphocyte count at admission were associated with elevated PNI levels (*β* = 0.077, *p* = 0.028; *β* = 0.315, *p* < 0.001; *β* = 0.009, *p* < 0.001; *β* = 1.359, *p* < 0.001). Patients with anemia before chemotherapy exhibited PNI scores 0.637 lower than those without anemia (*β* =  − 0.637, *p* = 0.005). In comparison with the patients receiving the TP regimen, those treated with specific chemotherapies (BEP, IP, GP, or FOLFOX) tended to present lower PNI scores (*β* =  − 1.188, *p* = 0.003). Increased defecation frequency per week was associated with higher PNI levels (*β* = 2.228, *p* = 0.029; *β* = 2.416, *p* = 0.019).

## Discussion

The detrimental impact of malnutrition on the clinical outcomes of cancer patients, encompassing complications and survival rates, has been extensively documented. However, the dynamic changes in nutritional status during multi-cycle chemotherapy in ovarian cancer (OC) patients have not been thoroughly explored.

To our knowledge, this study represents the first longitudinal examination where nutritional status served as a primary endpoint to ascertain the prevalence of nutritional risk. Our investigation tracked the three-wave dynamic changes in nutritional and hematological status during chemotherapy among OC women, revealing a sustained decrease in the prevalence of nutritional risk and a continuous improvement in nutritional status from chemotherapy initiation to the completion of cycles.

In our study, the incidence of nutritional risk before initial chemotherapy, and at the first, third, and fifth cycles were 17.0%, 14.7%, 7.4%, and 5.1%, respectively. This downward trend in nutritional risk prevalence among OC women as chemotherapy progressed is noteworthy. Bian et al. [21] reported a similar prevalence of nutritional risk (21.0%) before chemotherapy in cancer patients. The ascending trend observed in our study aligns with research by Gupta et al. [12], who found that the malnutrition prevalence decreased from 53.1% at admission to 35.7% at 3 months in OC women following chemotherapy, indicating an improved nutritional status. However, studies demonstrated a stabilization in the nutritional status of recurrent ovarian cancer women with intraperitoneal chemotherapy [22] and ovarian cancer women with post-operative chemotherapy [23]. Several potential reasons may explain these discrepancies in nutritional risk prevalence. First, diverse measurement tools to assess nutritional risk may contribute to inconsistent results. Second, cultural perspectives on eating habits and dietary structure vary across regions and countries, influencing nutritional status based on cultural background. Third, differences in tumor types, tumor locations, clinical stages, and lymphatic metastasis could also play a role.

Simultaneously, the variation trend of nutritional parameters aligned with that of nutritional risk, both indicating an improved nutritional status. A previous study demonstrated improvements in serum albumin levels and Mini Nutritional Assessment scores in patients with malignant lymphoma after chemotherapy [24]. In contrast, Movahed et al. [25] and Liang et al. [26] observed significant reductions in the levels of total protein and serum albumin in esophageal cancer patients during chemotherapy. Yamano [27] reported significant decreases in BMI and serum albumin levels in rectal cancer patients during and after chemoradiotherapy, with no significant difference in prealbumin levels.

The values of PNI decreased from 51.0 to 38.0 after treatment in head and neck cancer patients undergoing chemoradiotherapy (*p* < 0.05) [28]. Similarly, PNI decreased in 94.7% of breast cancer patients after chemotherapy, with a significant drop from 52.6 ± 3.8 pre-NAC to 46.5 ± 4.4 post-NAC. Our study, however, indicated sustained good levels of nutrition in OC women undergoing chemotherapy. According to Onodera et al. [13], the PNI normal value range is 50–60, while 40 is usually considered the cut-off point of malnutrition.

Several reasons contribute to the observed improvement in nutritional status. Chemotherapy typically commences only after reaching a certain BMI and blood test level to manage potential toxicities, emphasizing the importance of a healthy diet for OC women. The dropout rate in our study, with 95 patients dropping out, might exclude severely nutritionally impaired OC women, introducing bias to the results. Nonetheless, nutritional parameter levels decreased after chemotherapy, mainly due to the toxic side effects such as liver and kidney toxicity, and gastrointestinal symptoms. However, these effects were insufficient to alter the upward trend in nutritional parameters during chemotherapy cycles.

The outpatients might receive nutritional counseling or oral nutritional supplements (ONS) during chemotherapy treatment, which were overlooked in this study. Randomized controlled trials have indicated that nutritional counseling increases total energy, protein intake, and body weight, and improves overall survival in cancer patients undergoing chemotherapy [29, 30]. The use of oral nutritional supplements (ONS) is recommended when current dietary intake fails to meet nutritional requirements in cancer patients, in addition to normal food, with the recognized benefits of potentiating immune function, improving quality of life, and decreasing the incidence of adverse reactions during chemotherapy [31].

Considering the lower level of post-chemotherapy PNI compared with before chemotherapy due to the side effects of chemotherapy, we selected post-chemotherapy PNI as our independent variable. Investigating influencing factors of PNI aims to develop more targeted nutrition strategies to improve the prognosis of OC women undergoing chemotherapy.

This study identified pre-chemotherapy serum albumin, prealbumin, and absolute lymphocyte count as independent predictors of post-chemotherapy PNI. Higher pre-chemotherapy levels of serum albumin, prealbumin, and absolute lymphocyte count indicated higher post-chemotherapy PNI, aligning with previous findings [19]. Chen et al. [19] reported a significant association between high PNI and elevated levels of albumin and lymphocytes in breast cancer patients during chemotherapy. Serum albumin and prealbumin are traditional nutritional and inflammatory markers, while lymphocyte count reflects immune status. Low levels of serum albumin and lymphocytes can promote inflammatory tumor development and cancer spread [32, 33]. Prealbumin, a sensitive marker for malnutrition, holds prognostic importance in OC women [34]. For women with hypoalbuminemia and lymphopenia, pre-chemotherapy nutrition support with monitoring of albumin and lymphocyte levels is warranted.

Furthermore, as a nutritional indicator, body mass index (BMI) is related to cancer patient prognosis. This study reveals that pre-chemotherapy BMI is an influencing factor of PNI in OC women. Higher pre-chemotherapy BMI is associated with higher post-chemotherapy PNI, indicating a better prognosis. This finding is consistent with most previous studies [35–37]. Underweight patients (BMI < 18.5 kg/m^2^) had worse overall survival than non-underweight patients for all stages of colorectal cancer [35]. However, the role of BMI in different cancers may vary based on disease stage, tumor site, and treatment regimen, as demonstrated by Shepshelovich et al. [38], who showed that both underweight and morbidly obese statuses are associated with poor lung cancer survival. Moreover, a systematic review identified that the underweight status (BMI < 18.5 kg/m^2^) did not significantly influence ovarian cancer progression [39].

In this study, we revealed that pre-chemotherapy anemia is a potential risk factor for PNI, indicating that OC patients are more susceptible to malnutrition and worse outcomes when anemic before chemotherapy. This result aligns with previous studies [40–42]. Moreover, we observed a continuous reduction in hemoglobin levels over time during chemotherapy, contrasting with findings by Movahed et al. [25] and Liang et al. [26]. The multi-factorial and complex mechanisms of cancer anemia, including malnutrition, make it a recognized independent prognostic factor for poor patient survival [43]. A systematic review of 60 studies demonstrated a 65% higher overall risk of mortality in cancer patients with anemia compared to those without anemia [44]. The impact of anemia on prognosis may be linked to reduced quality of life, and delayed or incomplete chemotherapy regimens. Effective strategies targeting specific etiologies are essential to prevent and treat anemia of OC women.

Compared to patients with the TP regimen, those treated with specific chemotherapy types (BEP, IP, GP, or FOLFOX) tended to have lower PNI scores. Differences in chemotherapy regimens across disease stages and histological types of OC contribute to intrinsic inconsistencies of PNI. Additionally, higher daily defecation frequency per week was associated with higher PNI levels, suggesting a potential link between dietary intake, structure, and nutritional status during chemotherapy. Future research should focus on the relationship between variation in dietary intake and structure and nutritional status.

Despite the valuable insights gained, the study had limitations. The sample size, constrained by the single-center design, may limit result generalization. Variations in nutrition risk and status based on tumor stage, pathological type, location, and chemotherapy regimen warrant separate investigations. The lack of follow-up data on overall survival, complications, and other clinical outcomes necessitates exploration in future research. Additionally, the focus on nutritional status and risk during chemotherapy calls for future studies examining post-chemotherapy nutritional status over several months. Lastly, the rate of lost to follow-up in the present study was somewhat high, which could affect the accuracy of the analysis.

## Conclusions

Patients consistently demonstrated improvements in nutritional risk and status from the initiation to the completion of chemotherapy cycles. Nutritional monitoring of OC women, particularly those exhibiting abnormalities at the commencement of chemotherapy, is crucial. Targeted nutritional support programs should be developed to enhance the prognosis of OC women.

### Supplementary Information

Below is the link to the electronic supplementary material.

Supplementary file1 (DOCX 24 KB)

## Data Availability

No datasets were generated or analysed during the current study.
