# Supplementary material for: Trends in nutritional status and factors affecting prognostic nutritional index in ovarian cancer patients during chemotherapy: a prospective longitudinal study based on generalized estimating equations
Source: Support Care Cancer. 2024 Feb 24;32(3):191. doi: 10.1007/s00520-024-08384-8 (PMC10894134; doi:10.1007/s00520-024-08384-8)
Supplement: Supplementary file 1 — Supplementary file1 (DOCX 24 KB) [file 520_2024_8384_MOESM1_ESM.docx]

Table 1 Baseline Characteristics of Participants Present vs. Lost to Follow-up

| Variables | No. (%) | | *χ^2^ / t* | *P* value |
| --- | --- | --- | --- | --- |
|  | Present at follow-up (N = 525) | Lost to follow-up group (N = 95) |  |  |
| Age (year) |  |  |  |  |
| Means ± SD^a^ | 52.85 ± 10.67 | 51.48 ± 12.07 | 2.921 | 0.088 |
| ≤35 | 33 (6.3) | 8 (8.4) | 0.594 | 0.743 |
| 36-64 | 413 (78.7) | 73 (76.8) |  |  |
| ≥65 | 79 (15) | 14 (14.7) |  |  |
| Nation |  |  |  |  |
| Han | 514 (97.9) | 92 (96.8) | 0.071 * | 0.790 |
| other | 11 (2.1) | 3 (3.2) |  |  |
| Marital status |  |  |  |  |
| Married | 454 (86.5) | 83 (87.4) | 1.054 ** | 0.724 |
| Unmarried | 20 (3.8) | 5 (5.3) |  |  |
| Divorced | 24 (4.6) | 4 (4.2) |  |  |
| Widowed | 27 (5.1) | 3 (3.2) |  |  |
| Educational level |  |  |  |  |
| Primary school or below | 138 (26.3) | 15 (15.8) | 8.812 | 0.066 |
| Junior high school | 151 (28.8) | 26 (27.4) |  |  |
| Senior high school | 95 (18.1) | 18 (18.9) |  |  |
| Junior college | 78 (14.9) | 16 (16.8) |  |  |
| Undergraduate or above | 63 (12.0) | 20 (21.1) |  |  |
| Habitation |  |  |  |  |
| Rural | 92(17.5) | 15 (15.8) | 0.233 | 0.890 |
| Town | 136 (25.9) | 24 (25.3) |  |  |
| Urban | 297 (56.6) | 56 (58.9) |  |  |
| Per capita monthly income (yuan) |  |  |  |  |
| ≤630 | 16 (3.0) | 1(1.1) | 3.337 ** | 0.522 |
| 631～1000 | 40 (7.6) | 9 (9.5) |  |  |
| 1001～3000 | 133 (25.3) | 24 (25.3) |  |  |
| 3001～5000 | 229 (43.6) | 36 (37.9) |  |  |
| 5001～10000 | 81 (15.4) | 18 (18.9) |  |  |
| ＞10000 | 26 (5.0) | 7 (7.4) |  |  |
| Complication of diabetes |  |  |  |  |
| Without | 479 (91.2) | 89 (93.7) | 0.626 | 0.429 |
| With | 46 (8.8) | 6 (6.3) |  |  |
| Intestinal resection |  |  |  |  |
| Without | 443 (84.4) | 87 (91.6) | 3.359 | 0.067 |
| With | 46 (15.6) | 8 (8.4) |  |  |
| Chemotherapy regimens ^b^ |  |  |  |  |
| TP | 151 (28.8) | 27 (28.4) | 0.676 | 0.713 |
| TC | 332 (63.2) | 58 (61.1) |  |  |
| Others | 42 (8.0) | 10 (10.5) |  |  |

* Adjusted chi-square analysis

** Fisher’s exact probability method

Table 2 Clinical characteristics of the study population varied over time

| Characteristics | No. (%) | | | *χ^2^ / H* | *P* value |
| --- | --- | --- | --- | --- | --- |
|  | T1 | T2 | T3 |  |  |
| Change in dietary intake ^a^ |  |  |  |  |  |
| Unchanged | 420 (80.0) | 470 (89.5) | 495 (94.3) | **60.415** | < 0.001 |
| Increase (≤50%) | 38 (7.2) | 29 (5.5) | 16 (3.0) |  |  |
| Increase (＞50%) | 11 (2.1) | 6 (1.1) | 3 (0.6) |  |  |
| Reduce (≤50%) | 37 (7.0) | 12 (2.3) | 5 (1.0) |  |  |
| Reduce (＞50%) | 19 (3.6) | 8 (1.5) | 6 (1.1) |  |  |
| Average daily sleep duration (h) |  |  |  |  |  |
| ≥7 | 322 (61.3) | 336 (64.0) | 343 (65.3) | 1.880 | 0.391 |
| ＜7 | 203 (38.7) | 189 (36.0) | 182 (34.7) |  |  |
| Defecation frequency(times/week) |  |  |  |  |  |
| 0-3 | 19 (3.8) | 11 (2.2) | 10 (2.0) | 7.718 | 0.102 |
| 4-7 | 322 (63.8) | 350 (69.2) | 356 (70.4) |  |  |
| ＞8 | 164 (32.5) | 145 (28.7) | 140 (27.7) |  |  |
| Regular bowel movement |  |  |  |  |  |
| With | 84 (16.5) | 45 (8.8) | 48 (9.4) | **18.219** | < 0.001 |
| Without | 426 (83.5) | 465 (91.2) | 465 (90.6) |  |  |
| Average daily activity steps |  |  |  |  |  |
| ＜4400 | 191 (36.5) | 246 (46.9) | 250 (47.6) | **16.880** | 0.002 |
| 4400～7500 | 268 (51.1) | 221 (42.1) | 217 (41.3) |  |  |
| ＞7500 | 65 (12.4) | 58 (11.0) | 58 (11.0) |  |  |
| Chemotherapy-induced nausea ^b^ |  |  |  |  |  |
| Grade < 2 | 318 (60.6) | 352 (67.0) | 364 (69.3) | **9.618** | 0.008 |
| Grade ≥ 2 | 207 (39.4) | 173 (33.0) | 161 (30.7) |  |  |
| Chemotherapy-induced Vomiting ^c^ |  |  |  |  |  |
| Grade < 2 | 409 (77.9) | 430 (81.9) | 431 (82.1) | 3.765 | 0.152 |
| Grade ≥ 2 | 116 (22.1) | 95 (18.1) | 94 (17.9) |  |  |
| KPS score ^d^, median (IQR) | 90 (90,90) | 90 (90,90) | 90 (90,90) | 5.746 ^&^ | 0.057 |
| Barthel index, median (IQR) | 90 (90,95) | 90 (90,95) | 90 (90,95) | 2.907 ^&^ | 0.234 |
| In-hospital nutritional support ^e^ |  |  |  |  |  |
| Without | 473 (90.1) | 493 (93.9) | 502 (95.6) | 13.256 | 0.001 |
| With | 52 (9.9) | 32 (6.1) | 23 (4.4) |  |  |
| In-hospital parenteral nutritional support |  |  |  |  |  |
| Without | 497 (94.7) | 512 (97.5) | 509 (97.0) | **6.881** | 0.032 |
| With | 28 (5.3) | 13 (2.5) | 16 (3.0) |  |  |
| In-hospital enteral nutritional support |  |  |  |  |  |
| Without | 502 (95.6) | 506 (96.4) | 517 (98.5) | **7.477** | 0.024 |
| With | 23 (4.4) | 19 (3.6) | 8 (1.5) |  |  |
| In-hospital blood transfusion |  |  |  |  |  |
| Without | 520 (99.0) | 521 (99.2) | 522 (99.4) | 0.491* | 0.771 |
| With | 5 (1.0) | 1. (0.8) | 3 (0.6) |  |  |

* Fisher’s exact probability method

^&^ Kruskal-wallis H test

^a^ Change in dietary intake during the preceding month.

^bc^ Toxicity was graded according to Common Terminology Criteria for Adverse Events (CTCAE, v5).

^d^ Karnofsky performance scale, KPS.

^e^ Parenteral and enteral nutritional support, including intravenous infusion of albumin, amino acids, etc., and nutrition intake of protein powers, nutrient powders, and other fortified complementary foods.
